# Discovery of the Letheon

**Published:** 1847-08

**Authors:** 


2. *The Connecticut Legislature,* we are told, have passed a resolution declaring that Dr. Horace Wells, of Hartford, was the original discoverer of the inhalation of ether for surgical purposes. In a paper in the Boston Jour., Jan. 30th, entitled "A Review of Dr. M. Gay's Statement of Dr. C. T. Jackson's Claims, &c." by J. B. S. Jackson, M.D., of Boston, it is acknowledged that Dr. W., in Nov., 1844, did use the sulph.

eth., but abandoned it for the nitrous oxide, but this was two years subsequently to a conversation of Dr. C. T. J., with a Mr. Bemis and others on the subject of etherial inhalation. Even if established, as it seems to be, Dr. Wells' claim amounts to very little; and we advise him to abandon it for the future, and be content with the credit of good intentions. By *his* means, certainly, ether would never have come into general use. Mr. Morton's claims to any *discovery in this matter*, are satisfactorily shown to be wholly null and void. What pleases us most, is that the author of the article in the Journal says that Dr. J. "signed the petition for the patent in an unfortunate moment, and for legal reasons alone—that he deeply regrets ever having had anything to do with the patent"—so do we—that "he has never received any pecuniary advantage from it and never will," and that he has destroyed the bond given to him, and according to which Mr. M. was to have paid him a certain per centage of the profits which might be derived from the sale of the patent right. This is as it should be, and we honor Dr. Jackson for the course he has taken. The man who confesses and repents of a fault, has made for it the only reparation in his power, and deserves to be forgiven. We hope that such may be the end of every attempt at the violation of professional decorm, if such must be made: and that it may be long ere we have occasion to chronicle such another confession and recantation on the part of a man of so high standing in the scientific ranks of his countrymen. Mr. Morton the speculative adopter of other men's ideas, shorn of the falsely claimed honor of discovery, and disappointed of gain in the supply of all the world with the "patent" "compound" "letheon," stands alone in his glory! and there we leave him!—*Annalist.*

